# Effect of yeast culture on milk production and metabolic and reproductive performance of early lactation dairy cows

**DOI:** 10.1186/1751-0147-51-32

**Published:** 2009-08-03

**Authors:** Piret Kalmus, Toomas Orro, Andres Waldmann, Raivo Lindjärv, Kalle Kask

**Affiliations:** 1 Department of Therapy, Institute of Veterinary Medicine and Animal Science, Estonian University of Life Sciences, Tartu, 51014, Estonia; 2 Department of Environment and Animal Health, Institute of Veterinary Medicine nad Animal Science, Estonian University of Life Sciences, Tartu, 51014, Estonia; 3 Department of Reproductive Biology, Institute of Veterinary Medicine and Animal Science, Estonian University of Life Sciences, Tartu 51014, Estonia; 4 Department of Infectious Diseases, Institute of Veterinary Medicine and Animal Science, Estonian University of Life Sciences, Tartu, 51014, Estonia

## Abstract

**Background:**

The main objective of this study was to estimate the effect of supplementation with *Saccaromyces cerevisiae (SC*) (Yea-Sacc^® ^1026) on milk production, metabolic parameters and the resumption of ovarian activity in early lactation dairy cows.

**Methods:**

The experiment was conducted during 2005/2006 in a commercial tied-house farm with an average of 200 milking Estonian Holstein Friesian cows. The late pregnant multiparous cows (n = 46) were randomly divided into two groups; one group received 10 g yeast culture from two weeks before to 14 weeks after calving. The groups were fed a total mixed ration with silages and concentrates. Milk recording data and blood samples for plasma metabolites were taken. Resumption of luteal activity was determined using milk progesterone (P_4_) measurements. Uterine bacteriology and ovarian ultrasonography (US) were performed and body condition scores (BCS) and clinical disease occurrences were recorded. For analysis, the statistical software Stata 9.2 and R were used to compute Cox proportional hazard and linear mixed models.

**Results:**

The average milk production per cow did not differ between the groups (32.7 ± 6.4 vs 30.7 ± 5.3 kg/day in the SC and control groups respectively), but the production of milk fat (*P *< 0.001) and milk protein (*P *< 0.001) were higher in the SC group. There was no effect of treatment on BCS. The analysis of energy-related metabolites in early lactation showed no significant differences between the groups. In both groups higher levels of β-hydroxybutyrate (BHB) appeared from days 14 to 28 after parturition and the concentration of non-esterfied fatty acid (NEFA) was higher from days 1–7 post partum (PP). According to US and P_4 _results, all cows in both groups ovulated during the experimental period. The resumption of ovarian activity (first ovulations) and time required for elimination of bacteria from the uterus did not differ between the groups.

**Conclusion:**

Supplementation with SC had an effect on milk protein and fat production, but did not influence the milk yield. No effects on PP metabolic status, bacterial elimination from the uterus nor the resumption of ovarian activity were found.

## Background

Metabolic health is the successful adaptation of the dairy cow for higher energy requirements and metabolic changes during early lactation [1,2]. Many strategies, such as direct supplementation of propylene glycol, undegradable starch and monensin, have resulted in a positive effect on glucose production, but feeding dietary fat or specific fatty acids have not demonstrated improved energy status [3-6].

Based on a growing concern over the use of antibiotics and other growth promoters in the animal feed industry, interest in the effects of microbial feed additives on animal performance has increased. Supplementation with yeast culture has been used for over six decades and milk production responses have been investigated by several researchers. In some studies, cultures have improved dry matter intake, milk yield, and milk composition [7-11] whilst other studies have found no significant impact [12-16]. The energy balance of dairy cows is itself a key regulator of reproductive performance, changing the overall metabolic status during the periparturient period, which can lead to a delay in the resumption of ovarian activity and uterine involution. It has been established recently that the prolongation of postpartum NEB is a factor associated with low reproductive performance in dairy cows [17-19]. Many studies have demonstrated that the magnitude of NEB is related to the interval to first ovulation [17,20]. Animals suffering from NEB will have reduced resistance, which can lead to uterine infections and affect the PP uterus cleansing [21].

The objective of the study was to observe if cows with better fibre digestion and higher production values, may also show improved concentrations of energy related metabolites such as BHB and NEFA. If this is so, those animals might have a better energy balance, which may positively influence uterus cleaning and earlier resumption of ovarian activity. Considering previously described studies, microbial feed additives could be those substances which can help dairy cows better adapt to lactation needs and improve reproductive performance.

## Methods

### Cows and feeding

The experiment was carried out between December 2005 and May 2006 on a commercial tied-house farm with an average of 200 milking Holstein Friesian cows. The 46 late pregnant multiparous cows were randomly divided into two groups before calving, and were housed in separate rows on the farm. All cows calved during a two month period. Ten grams of SC (Yea-Sacc^® ^1026, Alltech Biotechnology Center, Nicolasville, YK, USA) were hand-mixed with a small amount of concentrate and were fed daily to each cow from the experimental group before morning feeding, starting from two weeks before the expected calving date until 14 weeks after parturition. Ten grams of SC is the recommended dosage according to Yea-Sacc^® ^1026 instructions. The cows were fed the same total mixed ration (TMR) diet. The TMR consisted of a grass silage and concentrate mix. Four different silage batches were used during the experimental period (Table 1). To control for the effect of different silage batches on the treatment, the feeding times of the silage batches were included in the statistical models.

**Table 1:** Chemical composition of the silages fed during the study period.

**Parameter**	Silage 1	Silage 2	Silage 3	Silage 4
Feeding period *	1.	2.	3.	4.

Dry matter %	40.3	32.7	39	32.7

Crude protein% of DM	15.86	13.65	14.8	16.12

NDF % of DM	40.9	58,2	58	46.03

ADF % of DM	35.1	34.8	36.1	31.7

ME MJ/kg of DM	9.47	9.65	9.84	10.18

* Feeding period of different silages

Silage 1: 15.12.05–05.01.06

Silage 2: 06.01.06–11.02.06

Silage 3: 12.02.06–27.03.06

Silage 4: 28.03.06–20.05.06

### Milk production data and body condition score

During the first 90 days in milk (DIM), cow identification number, date of calving, daily milk yield and disease occurrence data were recorded. Cows were milked twice a day. Every second day daily milk yield of cows was measured by automatic milking system (Milkmaster, Delaval). The milking units were checked before commencing recording according to DeLaval instructions.

Milk production data, including kg of milk produced, percentage and kg of milk fat and milk protein were recorded by the Estonian Animal Recording Centre every second week until 14 weeks after parturition. Body condition scores (BCS) were recorded once per week from the day of calving until week thirteen PP by trained personnel using a 5-point scale (1 = thin and 5 = fat) as described by Edmondson [22].

### Collection of milk samples for progesterone analysis

Milk collection for progesterone (P_4_) analysis was collected twice a week (Monday and Thursday) starting from the second week PP until the thirteenth week. In order to avoid the effect of the time of milk extraction on the P_4 _concentration, samples were collected not later than 1 h after the morning milking [23]. Milk (10 – 15 ml) was collected by handstripping into plastic tubes containing potassium dichromate as a preservative. Samples were frozen at -18°C until P_4 _analysis. Before analysis, milk samples were left to stand at room temperature overnight to thaw. The following day samples were centrifuged and concentrations of P_4 _in the milk were measured by enzyme immunoassay (EIA) according to Waldmann [24]. The inter- and intra-assay coefficients of variation were < 10%. The limit of sensitivity using a 20 μl sample was 0.5 ng/ml. Resumption of luteal activity was defined as the first two consecutive measurements of P_4 _concentrations > 3 ng/ml. Prolonged anoestrus was determined when consistently low P_4 _concentrations were measured for at least 50 days. [25]

### Plasma metabolites

Coccygeal blood vessel samples for biochemical analysis were collected in heparinized Venoject glass tubes (Terumo Europe N. V., Leuven, Belgium) once per week during the first 92 days PP.

After immediate centrifugation (15 min at 1048 × g), approximately 5 ml of plasma was removed and stored at -20°C until analysis. An automatic multiparameter analyser for clinical chemistry (EOS Bravo; Hospitex Diagnostics s.r.l., Italy) was used for enzymatic determination of plasma β-hydroxybutyrate (BHB) and non-esterified fatty acids (NEFA) with a commercially available kits (Randox Laboratories Ltd, UK).

### Collection of uterine biopsy specimen for bacteriological examination

Each animal in the study was sampled bacteriologically using endometrial biopsies once per week, starting within 7 days PP. Biopsy specimen collection was terminated when at least two consecutive negative samples were reported. In animals that only had negative samples from the beginning of collection, sampling was terminated after 3 weeks PP. Endometrial samples were collected aseptically according to the techniques and methods described previously by Kask *et al*.[26]. Biopsy specimens were immediately placed in a thioglycolate medium for transport to the Unit of Veterinary Microbiology, Estonian University of Life Sciences, for bacteriological examination. Cultivations were made within 1.5 h after collection. Standard bacteriological procedures according to Bergey's Manual of Systematic Bacteriology [27] were employed.

### Ultrasonographic examination of ovaries

The ultrasound (US) equipment was a real time B-mode linear array scanner (Hondex HS-120, Honda Electronics Co., Ltd., Aichi, Japan), with a 7.5 MHz transducer. Prints from a videographic printer were obtained. Ultrasound recording commenced 10 days PP and was performed twice per week (Tuesday and Friday) until the start of regular ovarian activity. Follicular activity was monitored in the ovaries. The sizes of the largest follicle and corpus lutea (CL) were monitored and measured by freezing the images and using callipers. Based on the size measurements, follicular dynamics were estimated. Ovulation was judged to have occurred if the dominant largest follicle(s) monitored by US could not be detected at the next examination, corpus luteum development was seen during subsequent examinations, and this was confirmed by a subsequent increase in P_4 _concentration [26]. All structures in the ovaries of more than 2.5 cm in diameter, and persisted for more than 10 days, were considered to be cysts [28].

### Statistical analysis

Health data were analyzed by the Fisher Exact Test using the statistical software Stata 9.2 [29]. Polynomial linear random-intercept models were used to explore time trend differences in milk production data, metabolic parameters and BCS between the experimental and control groups. Cows were included as random intercepts and polynomials of time in increasing order from parturition in days (or in weeks for milk fat and protein models) and their interactions with treatment were added as fixed effects until significant. Overall time trend differences between groups were tested with the F-test. The silage ratio was controlled in every model, and cow lactation time was included if significant. In the milk fat and protein models, milk yield on the sample day was used as a significant covariate. As the time between sampling was not the same in all cows, an isotropic spatial exponential correlation structure was used for modelling serial correlations of repeated measurements at the within-cow level in models for the metabolic parameters. In models for BCS, milk constitutes and milk yield, a first-order autoregressive (AR1) correlation structure was used as the time between sample points remained constant. The model assumptions were verified by scatter and normality plots of standardized residuals and logarithmic transformation of BHB, NEFA, BCS, and milk fat kg were used. The NLME package [30] with statistical software R 2.5.0 [31] was used for fitting these polynomial linear random-intercept models.

The Cox proportional hazards model was used to explore group differences in the timing of first rise in milk progesterone concentration (in weeks from parturition) and the time when uterine bacteriological examinations were reported to be negative (in weeks from parturition). The statistical software Stata 9.2 [29] was used for these models.

## Results

### Clinical diseases and exclusions

Altogether seven animals were removed from the experiment during the study period. Left displaced abomasum (n = 3), rumen collapse (n = 1) and downer cow syndrome (n = 2) were diagnosed in the control group. One case of downer cow syndrome was diagnosed in the experimental group. Final group sizes were 22 in the experimental group and 17 in the control group.

### Milk production

**Figure 1:**
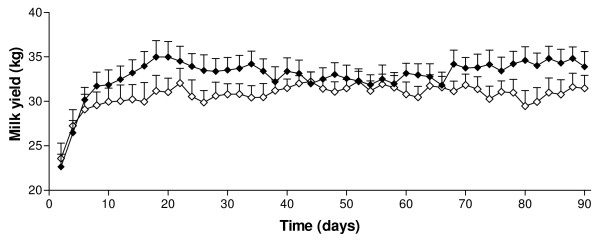
**Mean daily milk yield in Saccaromyces cerevisiae and control group**. Mean (± SEM) daily milk yield during the first 90 days from parturition (measured every second day) in cows from the Saccaromyces cerevisiae group (black diamond; n = 22) and control group (white diamond; n = 17).

**Figure 2:**
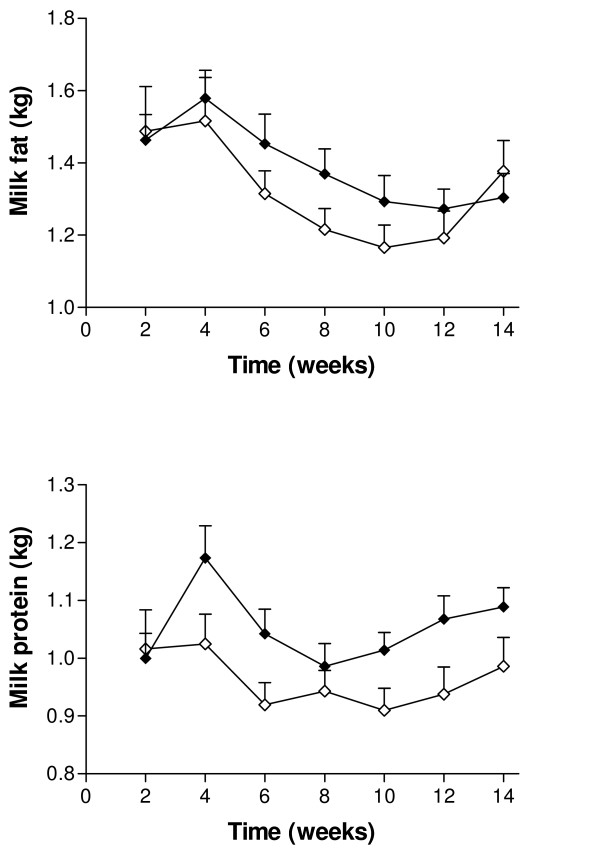
**Mean daily milk fat and protein production in Saccaromyces cerevisiae and control group**. Mean (± SEM) daily milk fat (above) and milk protein (below) during the first 14 weeks from parturition (measured every second week) in cows from the Saccaromyces cerevisiae group (♦; n = 22) and control group (◊; n = 17).

**Figure 3:**
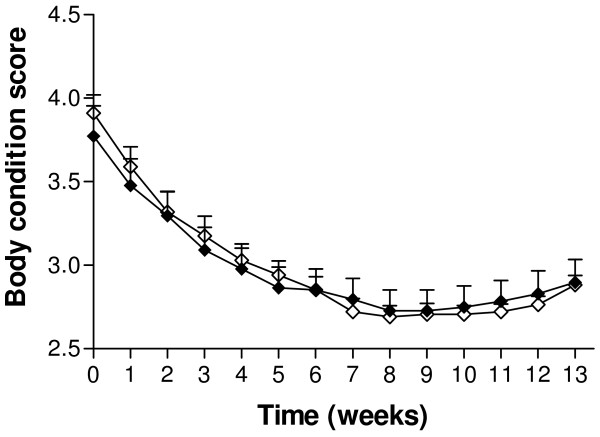
**Mean body condition score in Saccaromyces cerevisiae and control group**. Mean (± SEM) body condition score during the first 13 weeks from parturition (measured weekly) in cows from the Saccaromyces cerevisiae group (black diamond; n = 22) and control group (white diamond; n = 17).

**Figure 4:**
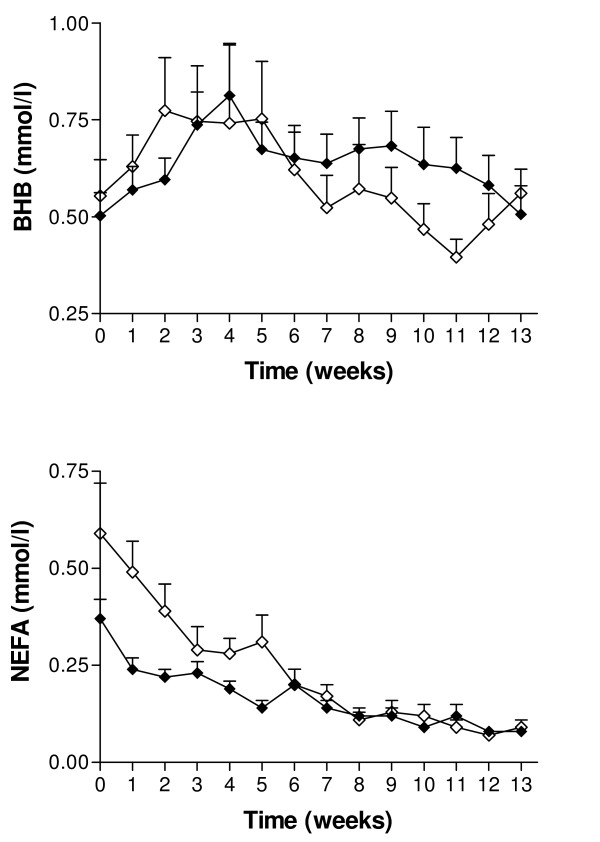
**Mean daily β-hydroxybutyrate and non-esterified fatty acid concentrations in the Saccaromyces cerevisiae and in the control group**. Mean (± SEM) daily β-hydroxybutyrate(BHB) concentrations (above) and non-esterified fatty acid (NEFA) concentrations (below) during the first 13 weeks from parturition (data pooled by sample week) in cows from the Saccaromyces cerevisiae group (♦; n = 22) and control group (◊; n = 17).

Supplementation with SC had no statistically significant effect on milk production over the study period. Mean (± SEM) daily milk yield was 32.7 ± 1.39 kg/d for the experimental group and 30.7 ± 5.3 kg/d for the control group. The changes in milk yield over time are illustrated in Figure 1. From 40 days PP, the milk yield in the two groups was similar and the curves did not differ significantly (*P *= 0.12). Both milk fat and protein production over time were significantly lower in the control group (*P *< 0.001 and *P *< 0.001, respectively; Figure 2). There was no effect of treatment on changes of BCS over time. A decrease in BCS was seen after parturition and the lowest scores were detected between days 56 and 63 PP in both groups (Figure 3). Figure 4 presents the changes in BHB and NEFA during the period up to 91 days PP.

Analyses of energy-related metabolites during early lactation showed no significant differences between the groups. An increase in BHB appeared from days 14 – 28 PP in both groups, where the decrease in NEFA stabilized on day 21 PP.

### Uterine bacteriology

In total, 234 uterine biopsies were collected from 39 cows, where 62 (26.5%) were found to have positive bacteriology results and the remaining 172 (73.5%) were negative. At the beginning of lactation, biopsies from 12 cows proved to be without bacterial growth. Of the 62 bacteriologically positive samples, 12 (19.3%) showed mixed infections and 50 (80.7%) had one of either aerobic or anaerobic cultures. The most frequently isolated anaerobic bacterium was *Fusobacterium necrophorum *(25%) whereas *Streptococcus *spp (30%) and *Arcanobacterium pyogenes *(22%) were the principal aerobic bacteria. The mean bacterial elimination time from the uterus was the third week in experimental group and the fourth week in the control group. No difference was found between the groups.

### Ovarian ultrasonography and P_4_

According to ovarian US, follicular activity was detected in all cows in both groups in the first US session on day 10 PP. The US and P_4 _results indicated that all cows in both groups ovulated during the experimental period. Prolonged anoestrus was detected in six cows (27%) in the experimental group and seven cows (41%) in the control group. Knowing that ovulation occurs approximately five days before this progesterone rise [25], the median (range) of resumption of luteal activity (first ovulations) was day 37 (14–93) in the experimental group and day 35 (9–112) in the control group. Before the onset of ovulation, regular ovarian activity (follicular waves with dominant follicle appearance and regression) was detected in both groups. The occurrence of ovarian cysts during the first four weeks PP was detected in five (22.7%) animals in the experimental group and four (23.5%) animals in the control group. There were no statistical differences between the groups.

## Discussion

### Milk yield and composition

Although not statistically significant, this equates to cows receiving SC having numerically higher (5.8%) milk yield than the controls. However, the sample size in the current study was very small to achieve significance for such numerical increases. Many studies have also reported an increase in milk yield, but again the effects have not been significant [32-36]. Some trials have noted a response to yeast supplementation only in early lactation cows [7,9,36]. Our study also showed that the largest difference between the groups appeared during the first six weeks PP.

Nocek et al. [37,38] reported increased milk fat and protein percentages when direct-fed microbial product was supplemented. We also found an impact of yeast supplementation on the milk protein and fat components, especially during early lactation. An explanation for the higher milk protein content in the experimental group could be the well-known impact of yeast on rumen fermentation and nutrient digestibility which enhances ammonia uptake and improves microbial protein production [8,39,40]. Increased milk fat percentage in very early lactation is often associated with adverse events such as excessive negative energy balance, rapid mobilization of body fats, and subclinical ketosis. However, neither mean blood BHB concentration nor the proportion of cows with elevated BHB concentrations increased in the experimental group. Similarly an effective digestion of fiber, in the form of neutral detergent fibre (NDF), will increase the number of cellulolytic bacteria in the rumen [41] and this could also influence milk fat content. For example, yeast supplementation had a significant effect on milk fat and protein content when NDF in the ration was 21% compared with 17% [42]. However, a lack of response in milk fat in many studies [8,12,13,15] could be indicative that the stimulation of fibre-digesting ruminal bacteria was sufficient for milk fat synthesis in those experiments.

### Metabolic parameters

Several factors including BCS, NEFA, the fat/protein ratio in milk, and ketone bodies have been found to be suitable parameters for indirect detection of energy balance. [21,43,44]. Milk from cows in the experimental group consisted of more protein and fat, which could indicate that stable rumen health may improve energy consumption PP and prevent serious metabolic changes. Elevated BHB levels suggest that fatty acids are being oxidized and that cows may be in a more severe state of negative energy balance [45], but no differences were found in our study. We found a higher concentration of NEFA around the time of calving in both groups, but subsequently stabilisation in concentrations was seen in the third week PP. The same findings have been described by other studies [37,38,46]

### Reproductive performance

Negative energy balance may affect ovarian activity by decreasing LH pulsativity, which leads delayed resumption of luteal activity [47]. Uterine infections have also been reported as risk factors for delayed ovulation [48]. To rule out endometritis as a possible cause for delayed ovulation, bacterial elimination time was investigated.

In our study, both groups showed a similar resumption of ovarian activity. Previous studies where direct-fed microbials [46,49] were used, also had no effect on reproductive function.

However, a large field study with a greater number of cows and herds is needed to determine the influence of yeast culture supplementation on reproductive performance.

## Conclusion

Based on the results of this investigation, supplementation with SC had an effect on milk protein and fat production, but did not influence the milk yield. No effect on PP metabolic status, bacterial elimination from uterus nor the resumption of ovarian activity were found.

## Competing interests

The authors declare that they have no competing interests.

## Authors' contributions

PK carried out the study, compiled the results and drafted the manuscript. TO participated in designing the study and statistical analysis of the data. AW participated in data collection and coordinated laboratory analysis, RL performed bacteriological analysis and KK coordinated the study. All authors were significantly involved in designing the study, interpreting data and composing the manuscript.
